# Low Threshold Vertical Cavity Surface Emitting Lasers Based on CsPbI_3_ Thin Films

**DOI:** 10.1002/advs.78163

**Published:** 2026-10-12

**Authors:** Naho Kurahashi, Maximilian Schiffer, Cedric Kreusel, Christian Tückmantel, Ahmed Kadid, Habib Ur Rehman, Florian Gaschler, René Anfang, Christopher Knoth, Maximilian Buchmüller, Patrick Görrn, Thomas Riedl

**Affiliations:** ^1^ Institute of Electronic Devices University of Wuppertal Wuppertal Germany; ^2^ Wuppertal Center for Smart Materials & Systems (CM@S) University of Wuppertal Wuppertal Germany; ^3^ Chair of Large Area Optoelectronics University of Wuppertal Wuppertal Germany

**Keywords:** amplified spontaneous emission (ASE), CsPbI_3_ thin film, optical gain, perovskite, vertical cavity surface emitting lasers

## Abstract

Metal halide perovskites are attractive gain media for compact coherent light sources. Although the all‐inorganic CsPbI_3_ is a particularly promising representative, low‐threshold laser operation remains challenging in CsPbI_3_ thin films due to phase instability and difficulties in preparing high‐quality thin films within low‐loss optical resonators. Here, we demonstrate the first vertical‐cavity surface‐emitting lasers (VCSELs) based on CsPbI_3_ thin films. The devices are fabricated via planar hot pressing using CsPbI_3_ thin films prepared through poly(vinylpyrrolidone)‐assisted phase stabilization, which enables simultaneous fabrication of smooth gain layers and well‐defined vertical cavities consisting of two distributed Bragg reflectors. Variable stripe length measurements on the CsPbI_3_ thin films are used for optical gain spectroscopy to guide the design of the VCSEL cavity mode by a rigorous coupled‐wave analysis. The optimized VCSELs afford optically pumped lasing at 720.4 nm coinciding with the maximum of the optical gain spectrum at a record‐low threshold energy density of only 2.8 µJ cm^−2^. Moreover, the VCSELs provide an outstandingly stable operation even under ambient conditions, which is attributed to the encapsulation inherently provided by the VCSEL structure. These results establish a pathway toward practical, low‐threshold perovskite VCSELs and highlight the potential of this platform for next‐generation laser devices.

## Graphical abstract

The first vertical‐cavity surface‐emitting laser (VCSEL) based on CsPbI_3_ thin films is fabricated by planar hot pressing. By optimizing the cavity design through optical gain assessment and rigorous coupled‐wave analysis, the VCSEL achieved an exceptionally low lasing threshold of 2.8 µJ cm^−2^. The device exhibits outstanding operational and ambient stability, benefiting from its low‐threshold operation and intrinsic architectural encapsulation.

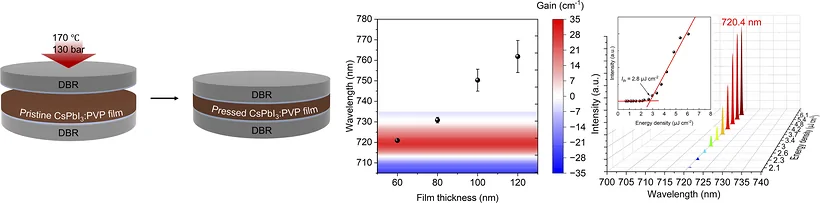

## 1 Introduction

Metal halide perovskites have emerged as highly promising materials for light‐emitting devices owing to their high photoluminescence quantum yield, tunable bandgap, and compatibility with low‐cost solution processing on diverse substrates [1, 2, 3]. Perovskite light‐emitting diodes (LEDs) now achieve external quantum efficiencies exceeding 25% across the visible to near‐infrared region [4, 5, 6, 7]. Furthermore, perovskites have attracted enormous interest as gain media for lasers [8]. Over the past decade, significant advances have been achieved in optically pumped perovskite lasers, including distributed feedback (DFB) and microcavity structures [9, 10, 11]. As a prerequisite for future electrical operation of a perovskite laser diode, LEDs capable of operating under high current injection have been realized [12, 13]. Moreover, perovskite LEDs have shown low‐threshold lasing under optical pumping, despite the presence of potentially lossy charge transport layers and electrodes [14, 15], as well as electrically assisted stimulated emission under combined optical and electrical excitation [16, 17]. Very recently, a perovskite vertical‐cavity surface‐emitting laser (VCSEL) optically pumped by an adjacent LED underscored the promising prospects of the VCSEL resonator configuration [18], which has culminated in the landmark demonstration of direct‐current‐driven polariton lasing in perovskite microplatelets integrated into a VCSEL at cryogenic temperatures [19]. Despite these remarkable milestones, bridging the gap towards stable, low‐threshold direct electrically driven operation at room temperature in scalable solution‐processed thin films remains a challenge. The threshold current densities estimated from optically pumped lasing are typically on the order of 10^2^–10^3^ A cm^−2^, exceeding the usual operation regime of conventional LEDs by orders of magnitude, which not only gives rise to enhanced nonradiative Auger recombination [20, 21] but also to Joule heating [22, 23], which in turn accelerates material degradation.

All‐inorganic cesium lead halide perovskites (CsPbX_3_, X = I, Br, Cl) exhibit superior thermal robustness compared to hybrid organic‐inorganic halide perovskites, making them attractive candidates for electrical operation. While major breakthroughs, including continuous‐wave (cw) operation [24, 25] and the first demonstrations towards direct electrical injection [19], have predominantly relied on CsPbBr_3_ and other green emitting compositions, the deep‐red emissive CsPbI_3_ (*E*_g_ ≈ 1.7 eV) is particularly appealing, as its emission spectrum is expected to show the least overlap with the absorption spectra of various charge transport layers, thereby reducing optical losses in future electrically driven device architectures [26, 27, 28, 29]. Compared with other red‐emitting perovskites, such as MAPb(I,Br)_3_ and Sn‐based perovskites, CsPbI_3_ combines an all‐inorganic composition with a Pb‐based iodide framework, avoiding both the thermal limitations associated with organic constituents and the susceptibility of Sn^2+^ to oxidation [30, 31]. Although the potential of CsPbI_3_ thin films as gain media has been severely compromised by their notorious phase instability, we have recently introduced poly(vinylpyrrolidone) (PVP) as an additive to overcome this bottleneck by maintaining the phase stability and we achieved a DFB laser based on CsPbI_3_ thin films, incorporating a linear grating directly patterned into the perovskite by thermal nanoimprint [32]. Importantly, PVP‐modified perovskites have also demonstrated efficient charge injection and performance in LEDs, indicating their compatibility with future electrical operation [33, 34].

Aside from the active gain medium, the choice of resonator architecture is crucial. Even though DFB lasers demonstrate low‐threshold lasing under optical pumping, their specific architecture can be expected to render electrical charge injection challenging [35]. In contrast to DFB resonators, VCSELs provide distinct structural advantages in this regard. Their vertically stacked short‐cavity geometry supports compact device architectures and, in optimized designs with highly reflective mirrors, provides a small optical mode volume that minimizes the total pump energy required for lasing [36], while low‐threshold operation requires careful minimization of cavity losses and appropriate overall cavity design. Furthermore, the vertical geometry shortens carrier transport paths and allows for charge injection geometries akin to conventional LEDs [37]. Despite the strong potential of CsPbI_3_ as gain medium and the advantageous properties of the VCSEL resonator architecture, no VCSEL based on CsPbI_3_ thin films has been reported to date. For solution‐processed perovskite thin films, VCSEL fabrication remains nontrivial, as issues regarding minimization of optical losses and the delicate matching of the optical cavity mode with the spectral gain characteristics arise. In polycrystalline films, surface roughness and grain boundaries introduce significant scattering and trap‐induced nonradiative recombination. This increases the required pumping levels for lasing and can also induce optical inhomogeneities that disturb stable cavity mode formation [38, 39]. These challenges make low‐threshold lasing in thin‐film‐based perovskite VCSELs more difficult than in their analogues based on single crystals, where reduced defect densities and improved optical uniformity are beneficial [40, 41, 42].

As halide perovskites can be considered soft materials, planar hot pressing (PHP), a variation of thermal nanoimprinting, provides an effective strategy to address the challenges imposed by polycrystalline thin films and enables the fabrication of perovskite thin films with improved morphology and crystallinity as well as significantly reduced defect density [32, 43, 44, 45].

Drawing from these unique beneficial properties, here we demonstrate for the first time low‐threshold lasing in VCSELs based on phase‐stabilized CsPbI_3_ thin films fabricated via PHP. To prepare a VCSEL, we leverage the opportunity to use the distributed Bragg reflectors (DBRs) as a substrate and a stamp to form a DBR/perovskite/DBR cavity [45]. Guided by a detailed study of the optical gain characteristics of CsPbI_3_ thin films, we conducted a rigorous coupled‐wave analysis to align the resonance of the vertical cavity with the maximum of the gain spectrum at ≈720 nm. The resulting VCSELs achieve low‐threshold lasing under pulsed optical excitation, while maintaining excellent operational stability even in ambient air. We expect our findings to inspire further developments toward the advancement of electrically driven perovskite lasers.

## 2 Result and Discussion

In the vertical cavity resonator architecture studied in this work, the perovskite gain medium is confined between two parallel DBRs (Figure 1a). Specifically, phase‐stabilized CsPbI_3_ films were deposited on the bottom DBR by spin‐coating a CsPbI_3_ precursor solution with PVP as an additive to form CsPbI_3_:PVP, following the approach reported in our previous work [32]. PHP was subsequently performed at 170°C with a pressure of 130 bar using an identical DBR as a stamp, which simultaneously functions as the top mirror of the resonator. More details about the PHP process can be found in the experimental section. To ensure spectral matching with the emission region of the CsPbI_3_:PVP, we employed commercially available DBRs (FD1C, Thorlabs) with a stopband spanning 575–785 nm (see Figure S1) and a reflectivity of up to 99.9% in the spectral window of 718–731 nm, relevant for the stimulated emission range of CsPbI_3_:PVP. For the characterization of the active medium after PHP, the top mirror has been carefully detached in some samples to expose the surface of the pressed CsPbI_3_:PVP layer. Top‐view scanning electron microscopy (SEM) images reveal that the CsPbI_3_:PVP films exhibit a notably smoothened surface after the PHP process (Figure S2). Note that we did not find any needle‐like features that are otherwise typically associated with the non‐perovskite yellow *δ*‐phase. X‐ray diffraction (XRD) measurements further confirmed that the CsPbI_3_:PVP films retain the perovskite black phase, with no sign of the yellow *δ*‐phase (see Figure S2). The photoluminescence (PL) spectrum of the films shows an emission peak centered at 698 nm, that falls within the stopband of the DBRs (Figure 1b). Note, the side peak at 644 nm, which appears exclusively for the film on the DBR, is likely attributed to the modified optical environment introduced by the DBR, rather than a material‐related phase (Figure S3).

**FIGURE 1:**
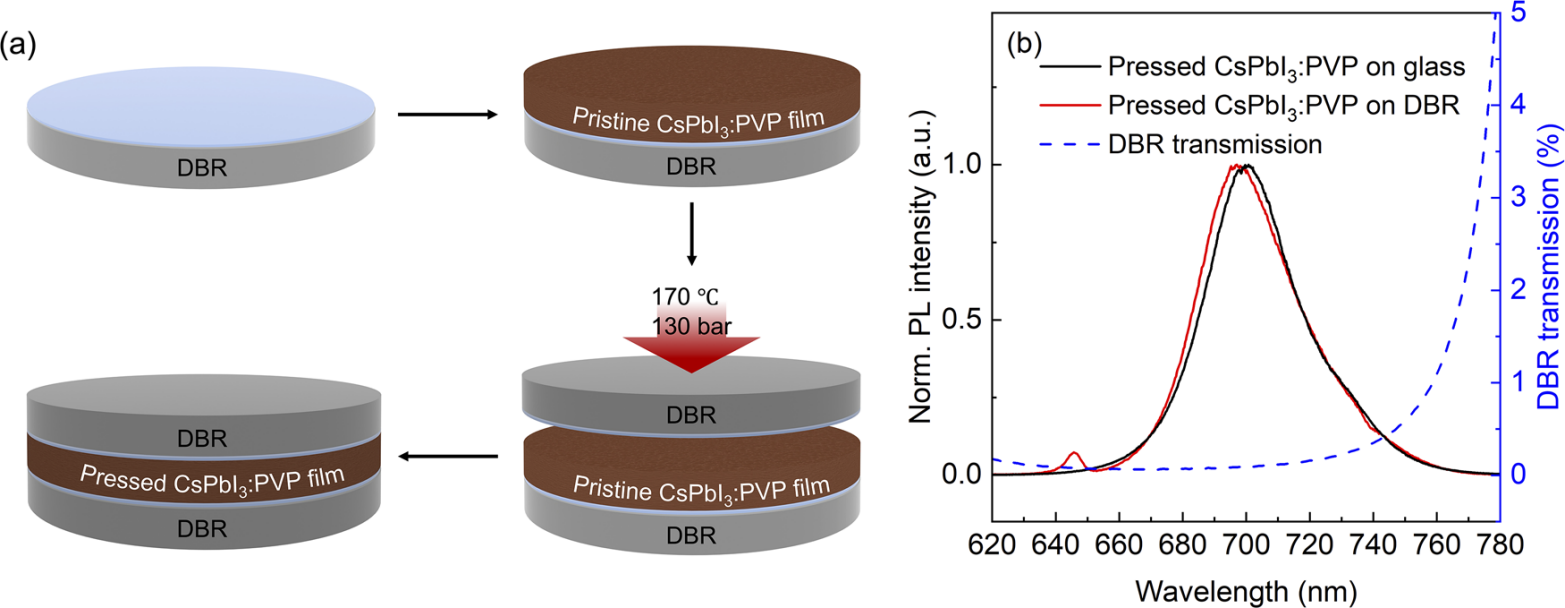
a) Schematic of the fabrication of a vertical‐cavity surface‐emitting laser (VCSEL) through planar hot pressing (PHP) using distributed Bragg reflectors (DBRs) (see text for details). b) Photoluminescence (PL) characteristics of the pressed CsPbI_3_:PVP film with a thickness of 100 nm created on a DBR as a substrate (red line) and on a glass substrate as a reference (black line). The transmission spectrum of the DBR is displayed as a dashed blue line. The full transmission spectrum of the DBR can be found in Figure S1.

The optical properties of the gain medium were further investigated under pulsed optical excitation (*λ*_3_ *_ω_* = 355 nm, pulse duration = 0.3 ns, repetition rate = 1 kHz). Figure 2a,b display the amplified spontaneous emission (ASE) characteristics of a 100 nm‐thick pressed CsPbI_3_:PVP thin film on top of a DBR as substrate. Above a threshold fluence (*I*_th_) of 71 µJ cm^−2^, a pronounced ASE peak centered at 724 nm with a substantially narrowed emission full width at half maximum (FWHM) of 4 nm emerges. Together with the ASE characteristics, the modal gain spectrum (*g*_mod_ (λ)) not only represents a crucial metric to evaluate the potential of a material for light amplification, but also provides essential guidance for the cavity design to align the resonance wavelength of the resonator with the maximum of *g*_mod_ (λ). Note that an analysis of the gain characteristics of CsPbI_3_ is missing, as of yet. We therefore characterized the modal gain characteristics of CsPbI_3_:PVP films using the variable stripe length (VSL) method [46]. For this purpose, a 100 nm‐thick CsPbI_3_:PVP film was deposited on Si/SiO_2_ substrates (SiO_2_ thickness of 1.5 µm) and was subsequently treated by PHP with a planar Si stamp under otherwise identical processing conditions as employed in our VCSELs. Note that we used Si/SiO_2_ substrates here because these allow for facile cleaving to prepare a clean sample edge, which is a key prerequisite for reliable VSL measurements. Likewise, the morphological improvement due to the PHP is highly beneficial for accurate gain characterization, as the VSL method operates in waveguide geometry and is therefore very sensitive to optical scattering in case of rough layers. As shown in the inset of Figure 2c, a pump laser beam focused to a stripe of variable length (*L*) was aligned perpendicular to the cleaved sample edge and the emission propagating along the excited stripe was collected at the sample edge. The spectrum was recorded as a function of *L* and the modal gain at each wavelength was extracted by fitting the emission intensity (*I*(*L*, *λ*)) using the established VSL formalism [46]:

$$I\left(L,\lambda \right)=\frac{I_{\mathrm{sp}}\left(\lambda \right)}{g_{\mathrm{mod}}\left(\lambda \right)}\left[e^{g_{\mathrm{mod}}\left(\lambda \right)\times L}-1\right]$$

**FIGURE 2:**
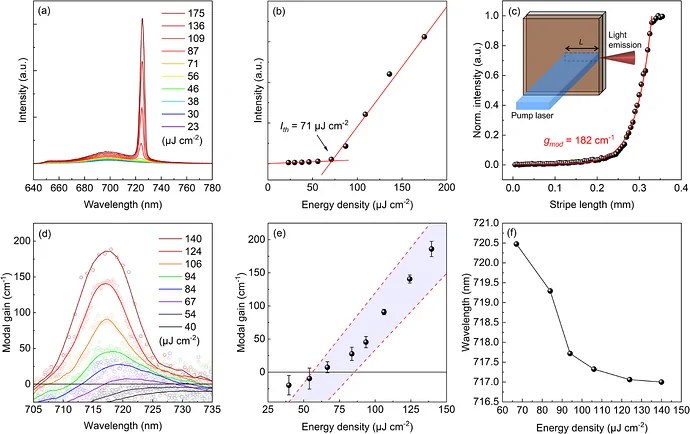
Amplified spontaneous emission (ASE) characteristics of a 100 nm‐ thick pressed CsPbI_3_:PVP film on top of the DBR: a) emission spectra and b) corresponding output characteristics with the determined threshold energy density. c) Edge emission intensity of a 100 nm thick pressed CsPbI_3_:PVP film excited using a variable stripe length (VSL) method at an excitation density of 140 µJ cm^−2^. A schematic of the VSL method is shown in the inset. d) Modal gain spectra of the 100 nm‐thick pressed CsPbI_3_:PVP film, e) corresponding gain slope with the shaded blue area representing the uncertainty associated with the linear fit, and f) shift in the spectral position of the maximum modal gain as a function of excitation energy density.

Here, *I*_sp_(λ) denotes a constant proportional to the spontaneous emission intensity. Figure 2c shows an example for *I*(*L*, λ) vs. *L* for the 100 nm‐thick pressed CsPbI_3_:PVP film at a wavelength of 717 nm and a pump energy density of 140 µJ cm^−2^. The exponential increase of *I*(*L*, λ) vs. *L* in this case indicates light amplification along the excited stripe as a result of positive optical gain experienced by the mode propagating in the waveguide formed by the perovskite. A similar evaluation for all *λ* in the spectral region of interest allows to determine the modal gain spectrum *g*_mod_ (λ). The fluence dependence of the gain characteristics in Figure 2d,e indicates that optical gain emerges around 720 nm at a fluence of around 56–86 µJ cm^−2^. The spectral gain maximum region coincides well with the resonance wavelength at which we found the lowest lasing threshold in DFB lasers based on CsPbI_3_:PVP [32]. The differential modal gain is estimated to be in the range of 2.3–2.7 cm µJ^−1^, which agrees with values inferred from fluence‐dependent modal gain measurements reported for other perovskites [47, 48, 49]. Note that the modal gain is defined as *g*_mod_ =  Γ *g*_mat_, where *g*_mat_ is the material gain and *Г* is the confinement factor that accounts for the spatial overlap of the guided optical mode with the gain medium [50]. By incorporating the *Г* of 0.55 estimated by a simple transfer matrix simulation for the 100 nm‐thick film (Figure S4), we derived a differential material gain of 4.2–4.9 cm µJ^−1^. The spectral position of the maximum of *g*_mod_ (λ) shows a clear blue shift of about 3 nm with increasing excitation fluence from 67 to 140 µJ cm^−2^ (Figure 2f). Simultaneously, as the excitation density increases, broadening of the gain spectrum is observed with a FWHM of 12 nm at fluence of 140 µJ cm^−2^, reflecting the wide spectral window over which the material is able to provide positive optical gain. These spectral changes suggest that the gain in our CsPbI_3_:PVP originates from free carriers rather than excitons [47]. Note that the exciton binding energy (*E*_b_) of our CsPbI_3_:PVP was estimated to be approximately 20 meV, resulting from an Elliott fitting of the absorption spectrum and Arrhenius fitting of the temperature‐dependent PL intensity (see Figures S5 and S6), which falls within the range of previously reported values for CsPbI_3_ obtained using temperature‐dependent optical spectroscopy and low‐temperature magneto‐transmission spectroscopy [51, 52, 53]. As *E*_b_ is smaller than the thermal energy at room temperature (*k*_B_ *T* = 26 meV) excitons readily dissociate under ambient conditions, generating a significant population of free carriers. In such a regime, optical gain arises predominantly from band‐to‐band transitions of free electrons and holes rather than excitons. These continuum transitions span a broad range of energies, in contrast to the narrow linewidth associated with excitonic recombination, and therefore account for the pronounced spectral broadening observed in the gain spectrum. A blue shift of the gain spectrum observed with increasing pump fluence is consistent with the free‐carrier model known from III–V semiconductors [54].

In the next step, we aimed to spectrally align the cavity resonance of our VCSELs with the spectrum of the optical gain by variation of the thickness of the CsPbI_3_:PVP gain layer (via adjustment of the precursor concentration in the spin‐coating process). Figure 3a shows the positions of the dominant resonance modes obtained from PL spectra of the resulting CsPbI_3_:PVP VCSELs (excited with a frequency‐tripled, continuous wave laser, *λ*_3_ *_ω_* = 355 nm, at low intensity of 4 W cm^−2^, i.e., significantly below the ASE threshold). Reducing the thickness of the gain medium from 120 to 60 nm affords a shift of the resonance wavelength by approximately 40 nm, and a 60 nm‐thick CsPbI_3_:PVP gain layer exhibited a cavity mode that provided the optimal spectral overlap with the gain maximum region. Such a large shift cannot be explained by a simple Fabry–Pérot cavity model, which assumes two well‐defined cavity boundaries without field penetration into the mirrors. In our case, however, the DBRs are composed of multilayer dielectric stacks, into which the optical fields can extend significantly. To account for this, we first reconstructed the full optical stack by reverse engineering the DBR layer stack from SEM cross‐sectional images and performed rigorous coupled‐wave analysis (RCWA) to simulate the optical response (Figure 3b) [55]. Each DBR consists of 50 alternating layers of SiO_2_ and Ta_2_O_5_, resulting in an overall VCSEL stack consisting of 101 layers including the perovskite gain medium. The final thickness of the optimized gain layer in the VCSEL was also verified by cross‐sectional SEM, confirming a uniform thickness of 60±1.6 nm (see Figure S7). To achieve agreement between simulation and experiment, we performed an additional constrained optimization step, in which the thicknesses of all 50 SiO_2_ layers were varied by a common factor, while the thicknesses of all 50 Ta_2_O_5_ layers were varied by another common factor. These variations in layer thickness were introduced to account for uncertainties in the layer thicknesses extracted from SEM cross‐sectional images, as well as for idealized assumptions made by the simulations such as perfectly smooth and homogeneous layers, as well as perfectly abrupt material interfaces. The refractive indices of both materials were kept fixed at their bulk values for all simulations. Using said thickness variations, we found well‐fitting simulated transmission spectra and resonance positions compared to the experimental data by only varying the layer thicknesses by less than 10%. Figures S8–S10 provide further details about the stack optimization and simulations. Our results confirm that the distributed nature of the cavity modes can indeed account for the observed resonance behavior and the shift in resonance positions upon perovskite thickness variations (Figure S9). As shown in Figure 3c, the optimized VCSEL with a 60 nm‐thick CsPbI_3_:PVP gain layer exhibits a sharp longitudinal mode at 720.4 nm with a FWHM of 0.65 nm within the high‐reflectivity DBR stopband, yielding a high cold cavity *Q* factor of ≈1.1×10^3^, which indicates low optical losses of the resonator.

**FIGURE 3:**
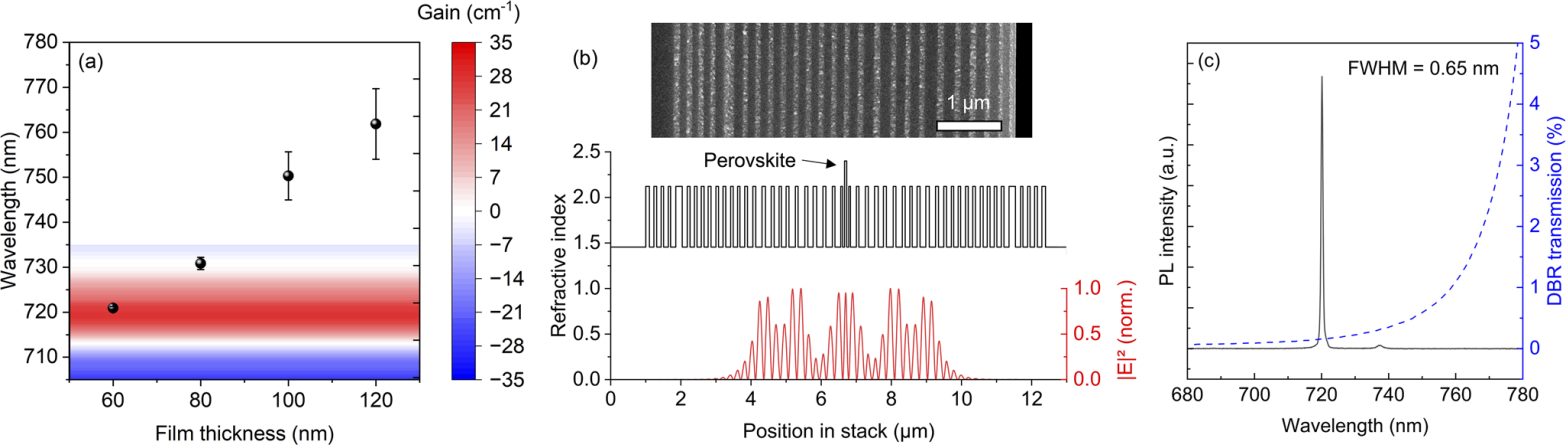
a) Cavity mode wavelength of CsPbI_3_:PVP VCSELs for varied perovskite film thickness. The gain spectrum obtained at an excitation density of 84 µJ cm^−2^ is overlaid as a guide to illustrate the spectral profile highlighting regions with positive optical gain. The color scale represents the modal gain coefficient. b) A cross‐sectional scanning electron microscopy (SEM) image of the DBR (top). Refractive index profile (black) and corresponding simulated electric field intensity distribution (red) of a VCSEL with a 60 nm‐thick CsPbI_3_:PVP layer sandwiched between two DBRs. Note that the mode penetrates significantly into the DBR layers, indicating that the effective cavity length extends far beyond the physical thickness of the CsPbI_3_:PVP gain medium. c) Cavity mode spectrum (below threshold) obtained from a VCSEL with a 60 nm‐thick CsPbI_3_:PVP layer. The transmission spectrum of the DBR is displayed as dashed blue line.

Having identified the 60 nm‐thick layer to afford a resonance that shows the best spectral coincidence with the maximum of the optical gain spectrum, we investigated the morphological and optical characteristics of these films. We focused our comparison on this optimized 60 nm‐thick layer and the initially studied 100 nm‐thick layer, for which the gain characteristics were established despite its detuned cavity resonance. Meanwhile, other thicknesses such as 120 and 80 nm were excluded due to suboptimal resonance matching with the gain spectrum, as illustrated in Figure 3a and detailed in Figure S11. Atomic force microscopy (AFM) analysis revealed that the 60 nm‐thick film has a lower roughness (0.8 nm, rms) compared to the 100 nm‐thick film (2.3 nm, rms) (Figure 4a,b). The reduced surface roughness is expected to further reduce scattering‐induced optical losses, thereby preserving the high optical quality of the cavity. To verify this, we evaluated the cold cavity spectrum of the VCSEL with a 100 nm‐thick CsPbI_3_:PVP layer, which exhibits a lower *Q* factor of ≈425 (Figure S12). The significant enhancement in *Q* factor observed for the VCSEL with the smoother 60 nm‐thick film (≈1.1×10^3^) correlates with the reduction in surface roughness, confirming that suppressing surface scattering plays a role in reducing optical losses. The PL spectra show an emission peak at ≈700 nm for both the pressed 60 nm‐ and the 100 nm‐thick film, while the 100 nm‐thick film exhibits a notable low‐energy shoulder that is absent in the 60 nm‐thick film (Figure 4c), which may be explained by a reduction in the density of trap‐mediated emission states [56]. Elliott fitting analysis of the absorption spectra indicates that the 60 nm‐thick film exhibits a narrower excitonic feature compared to the 100 nm‐thick film, suggesting reduced inhomogeneous broadening (Figure S5). This change is consistent with a sharper excitonic band‐edge and suggests reduced structural disorder, which is often associated with a lower density of sub‐bandgap states in perovskites [57, 58]. This is further corroborated by power‐dependent PL measurements (Figure S13). The PL intensity (*I*_PL_) as a function excitation density (*P*) follows a power‐law relationship, *I*_PL_ ≈ *P^k^*, with *k* = 1.55 for the 100 nm‐thick film, indicating that trap‐mediated non‐radiative recombination remains significant [59]. In contrast, the 60 nm‐thick film shows a transition from *k* = 1.50 to 2.06 as the excitation increases, implying that trap states are more readily filled in the 60 nm‐thick film, and radiative free‐carrier recombination dominates at higher carrier densities. In films without a defined optical cavity, stimulated emission is generally affected by not only the layer quality but also optical confinement. For the 60 nm‐thick film, the optical confinement factor *Г* is estimated to be 0.26 (Figure S4), which is significantly lower than that of the 100 nm‐thick layers. Although this reduction in *Г* compared to the 100 nm‐thick film leads to a somewhat higher ASE threshold, robust stimulated emission is maintained (Figure S14).

**FIGURE 4:**
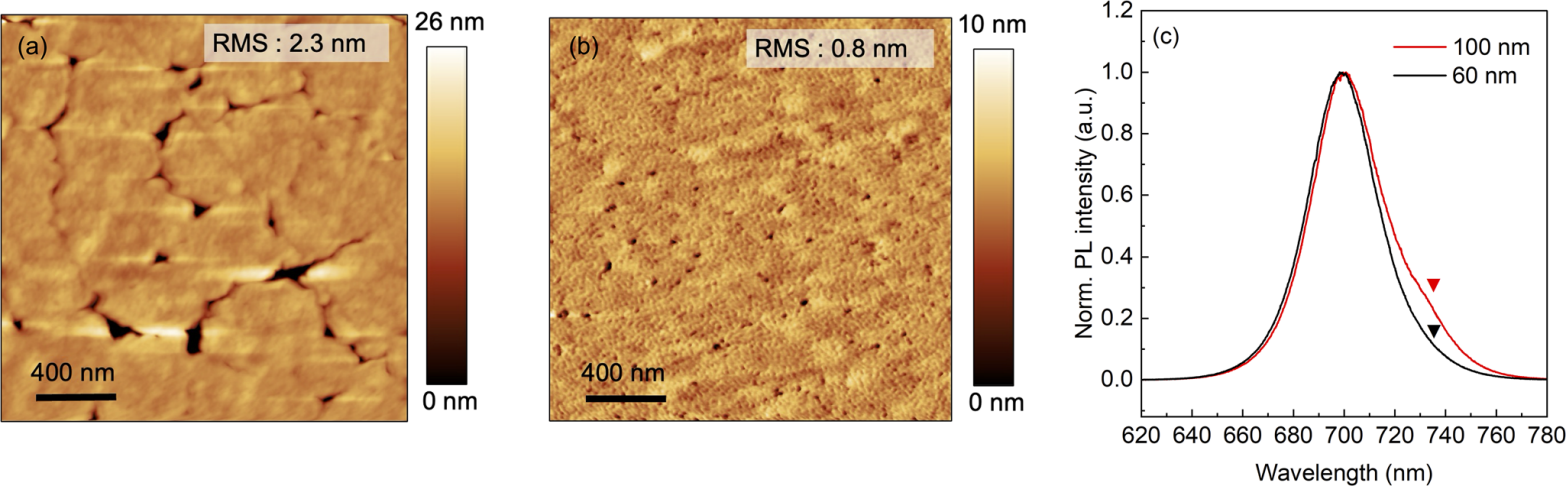
Atomic force microscopy (AFM) images of a) 100 nm‐ and b) 60 nm‐thick CsPbI_3_:PVP films after PHP. c) PL spectra of the corresponding films.

We next investigated the lasing characteristics of the VCSELs with the 60 nm‐thick CsPbI_3_:PVP gain layer under pulsed optical excitation. As the excitation fluence increased, a narrowed emission peak emerged at 720.4 nm (Figure 5a), and the emission intensity of the peak exhibited an abrupt increase above a threshold of 2.8 µJ cm^−2^, as shown in Figure 5b. The high‐resolution emission spectrum of the laser line in Figure 5c reveals a width of *Δλ* = 0.16 nm, which is close to the limit of the resolution of the spectrometer and significantly narrowed with respect to the width of the cavity mode emission spectrum below threshold of 0.65 nm (Figure 3c). While the 60 nm‐thick film exhibits a high ASE threshold of 230 µJ cm^−2^, the corresponding VCSEL achieves a remarkably low lasing threshold fluence of 2.8 µJ cm^−2^. This is exceptionally low for a solution‐processed perovskite laser, particularly considering the relatively long‐pulse excitation (0.3 ns) compared to ultrafast regimes (see Table S2). In contrast to ASE in the bare film, where no resonant optical feedback is present and amplification occurs over a finite in‐plane propagation length, the high‐Q VCSEL cavity provides resonant optical feedback that extends the photon lifetime and increases the intracavity photon density, thereby facilitating stimulated emission [60]. Similar reductions in threshold upon vertical cavity integration have been reported for other perovskite VCSELs, including our previous work on CsPbBr_3_ VCSELs [45, 61, 62]. In addition, the cavity resonance of the optimized VCSEL is spectrally aligned with the peak gain of the active layer, providing favorable conditions for low‐threshold lasing.

**FIGURE 5:**
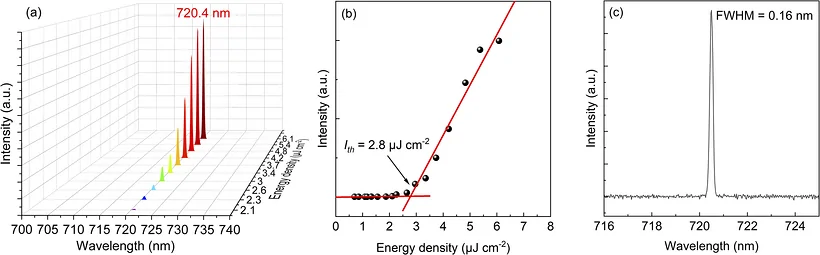
a) Emission spectra with increasing energy density and b) corresponding output characteristics of the CsPbI_3_:PVP VCSEL upon optical pumping with the determined lasing threshold. c) High‐resolution spectrum of the CsPbI_3_:PVP VCSEL lasing peak under pulsed excitation at a fluence 20% above the lasing threshold.

Finally, we evaluated the stability of the VCSELs with the 60 nm‐thick CsPbI_3_:PVP layer. Figure 6a shows the laser intensity under pulsed excitation at a fluence 50% above the threshold under ambient conditions. The VCSELs remained at more than 50% of their initial output intensity after 1.6 × 10^6^ excitation pulses, whereas a previously reported CsPbI_3_:PVP DFB laser lasted for only 1.4 × 10^5^ pulses, even under inert conditions [32]. This improvement is primarily attributed to the low lasing threshold of the VCSELs, which mitigates photo‐induced degradation under optical pumping [63]. Furthermore, the long‐term shelf stability was assessed over 30 days of storage in ambient air. As shown in Figure 6b, the VCSELs retained their low lasing threshold after 30 days of storage in air, demonstrating robust resistance to environmental degradation. This long‐term stability is largely attributed to the intrinsic encapsulation effect of the VCSEL configuration, which physically isolates the perovskite layer from direct exposure to oxygen and moisture.

**FIGURE 6:**
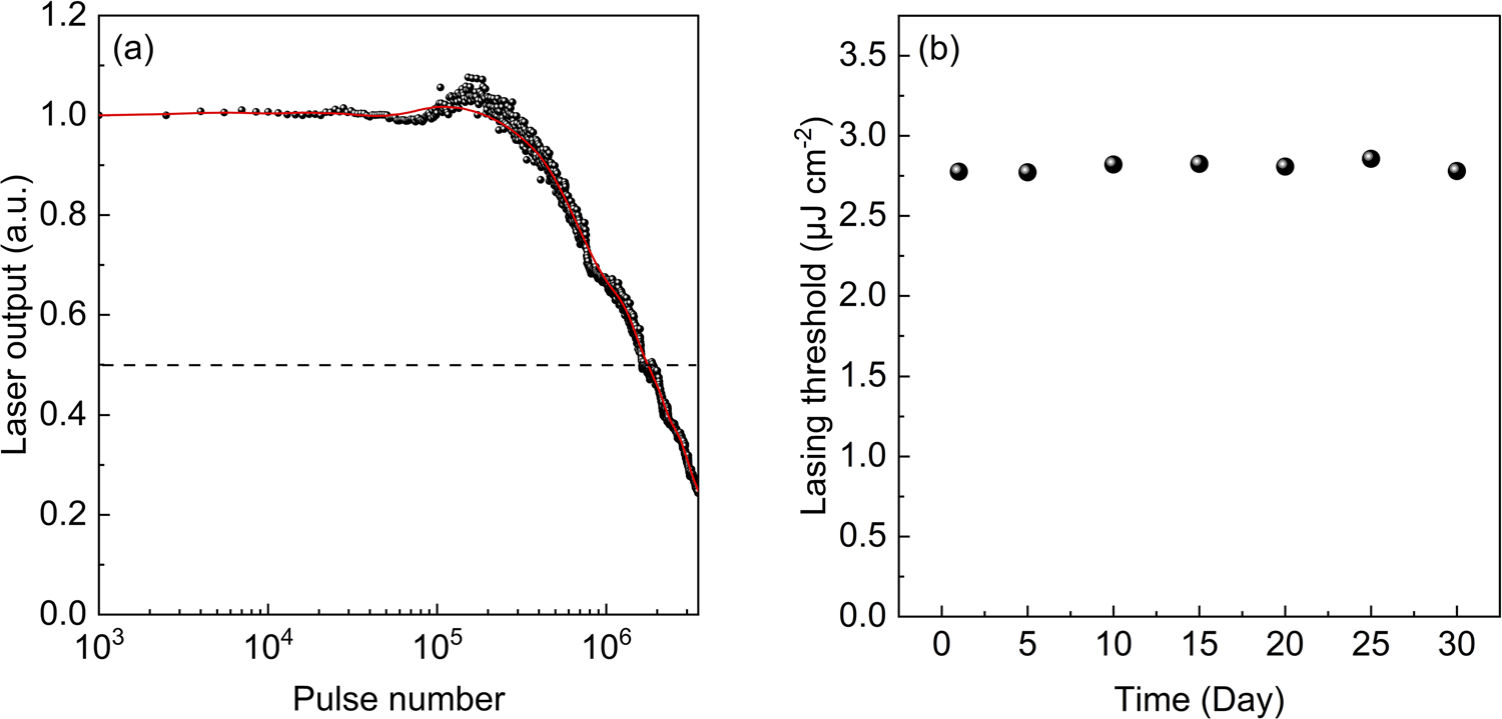
a) The laser output of the CsPbI_3_:PVP VCSEL as a function of number of excitation pulses. The red line provides a guide to the eye. b) Lasing threshold of the CsPbI_3_:PVP VCSEL monitored periodically during storage in air. Measurements were performed at room temperature under ambient conditions.

Looking ahead, the combination of solution‐processability, low lasing threshold, and environmental stability demonstrated in our CsPbI_3_:PVP VCSELs provides a promising platform for next‐generation coherent light sources. Towards cw and electrical operation, further optimization will be required to address the thermal limitations of standard SiO_2_/Ta_2_O_5_ DBRs, such as through the integration of alternative high thermal conductivity mirror systems [64, 65] or thermally conductive 2D layers such as hBN [66], alongside the development of electrically conductive DBRs, which remain largely unexplored for perovskite lasers.

## 3 Conclusion

In conclusion, we have demonstrated a VCSEL utilizing a phase‐stabilized CsPbI_3_ thin film integrated between DBRs. PHP enabled the simultaneous fabrication of a high‐quality gain medium and a vertical resonator in one step. Based on a careful assessment of the optical gain of our perovskite and using a rigorous coupled‐wave analysis, we optimized the cavity design to align the cavity resonant mode to coincide with the maximum of the optical gain spectrum. Thereby, we were able to realize low‐threshold VCSELs emitting at 720.4 nm, with a record‐low threshold of 2.8 µJ cm^−2^ under 0.3 ns pulsed excitation. Furthermore, the device exhibited excellent operational and ambient stability, attributed to the low‐threshold operation and the intrinsic encapsulation of the VCSEL architecture. We expect our findings to provide key insights for developing scalable coherent light sources and, ultimately, advancing electrically pumped perovskite lasers.

## 4 Experimental Section

### 4.1 Materials

Cesium iodide (CsI: ultra dry, 99.998% (metals basis)) and lead iodide (PbI_2_: ultra dry, 99.999% (metals basis)) were purchased from ThermoFisher. Anhydrous dimethylsulfoxide (DMSO: ≥ 99.9%) and poly(vinylpyrrolidone) (PVP: K30) were purchased from Sigma‐Aldrich. All chemicals were stored in an N_2_‐filled glovebox and used as received.

### 4.2 Preparation of Perovskite Films

CsI and PbI_2_ were dissolved in DMSO with a ratio of CsI to PbI_2_ of 1:1. The precursor was stirred overnight at 70°C and filtered using a 0.2 µm filter. The PVP was dissolved in the CsPbI_3_ solutions with a 5 wt% ratio with respect to CsPbI_3_ precursors. The mixture was stirred for 1 hour at 70°C and cooled to room temperature before use. Si, Si/SiO_2_, and glass substrates for the film characterization were cleaved 22 × 22 mm using a diamond cutter and cleaned by sonication in citric acid, deionized water, acetone, and isopropanol (each step 5 min). DBRs (FD1C, Thorlabs) were used as a substrate for the VCSEL fabrication. All substrates were activated with an excimer lamp for 3 min and immediately transferred to an N_2_‐filled glovebox. CsPbI_3_:PVP layers were prepared by spin‐coating the mixture solutions at 3000 rpm, 30 s, and 10 s ramp. After spin‐coating, the films were annealed on a hot plate at 160°C for 2 min. All the film fabrication process was carried out in an N_2_‐filled glovebox.

### 4.3 PHP and VCSEL Fabrication

For the Si, Si/SiO_2_, and glass samples, the flat Si stamps with a coated surface with an anti‐sticking layer [67] were used to smooth the surface of the spin‐coated film by the PHP process. For the VCSEL fabrication, DBRs were used as a substrate for the deposition of the perovskite, and an identical mirror was used as the stamp for the PHP process. The stack was sandwiched with thermally conductive rubber (KU‐CG20/R, Boyd) and aluminum foil. The samples were packed in an N_2_‐filled glovebox. A parallel plate‐based imprint system was used for the PHP process [68, 69]. Firstly, pressure was gradually applied until the pressure force reached 130 bar. Secondly, the pressure was kept constant for 30 min after the temperature reached the set value of 170°C. Finally, the pressure was released when the system was cooled down below 25°C by a water‐cooling system.

### 4.4 Characterization of Perovskite Films and VCSELs

The layers on Si substrates and DBRs were investigated by using an SEM (ZEISS Crossbeam 550). For XRD, a Bruker D2 phaser with a Cu Kα anode (*λ* = 1.54184 Å) combined with a Lynxeye (1D mode) detector was used to investigate layers on Si substrates. AFM images of the layers on DBRs were taken by using a Bruker Innova system (tapping mode at 300 kHz). For PL measurements, a continuous wave, diode pumped, frequency tripled solid state laser (*λ* = 355 nm) was used. The light emission from layers deposited on glass substrates and DBRs was coupled into a monochromator (Princeton Instruments, Acton SP2500, grating: 300 lines mm^−1^), and the spectrally dispersed light was detected by a thermoelectrically cooled charge‐coupled device camera (Princeton Instruments, PIXIS‐100). Optical gain measurements were carried out using the VSL method. A stripe‐shaped excitation spot was formed using cylindrical lenses and the stripe length was controlled by an adjustable slit mounted on a motorized translation stage that was operated via a LabVIEW‐controlled measurement system. Emission from the edge of cleaved Si/SiO_2_ samples was passed through a polarizer to select the TE‐polarized component, coupled into a monochromator (Acton SP2500, grating: 300 lines mm^−1^) and detected using a thermoelectrically cooled charge‐coupled device camera (Princeton Instruments, PIXIS‐100). The emission intensity as a function of stripe length was automatically transferred to a Python‐based analysis program. The data were fitted with the classic gain model to extract the modal gain coefficient as a function of wavelength. UV–Vis absorption measurements were conducted at room temperature on glass substrates within an integrating sphere. The thin films were illuminated by a collimated balanced deuterium tungsten halogen light source (DH‐2000‐BAL, Ocean Optics) in reflection (8° incident angle) and transmission geometry. The respective spectra were fiber‐coupled into a monochromator (Acton SP2500, grating: 150 lines mm^−1^) and detected by a thermoelectrically cooled charge‐coupled device camera (Princeton Instruments, PIXIS‐100). The absorption coefficients were derived from the Lambert‐Beer law. Temperature‐dependent PL measurements were performed in cryostat system (Janis CCS‐400, 8–500 K). The thin film on Si/SiO_2_ was optically pumped by a frequency‐tripled diode‐pumped solid‐state laser (PowerChip NanoLaser, TEEM Photonics, France) with *λ* = 355 nm. The temperature‐dependent PL intensity was analyzed using the Arrhenius equation to determine the exciton binding energy. For the ASE of the layers on the DBRs and VCSEL characterization, samples were optically pumped by a frequency‐tripled diode‐pumped solid‐state laser (PowerChip NanoLaser, TEEM Photonics, France) with *λ* = 355 nm, a pulse duration of 0.3 ns, and a repetition rate of 1 kHz. A neutral density filter wheel controlled the excitation density. The power of the laser source was measured with a photodiode power sensor (S130VC, Thorlabs). The laser spectra dispersed in a monochromator (Princeton Instruments, Acton SP2500, gratings: 300, 1200 lines mm^−1^) via a multimode optical fiber, and spectrally dispersed light was detected by a thermoelectrically cooled charge coupled device camera (Princeton Instruments, PIXIS‐100).

## Author Contributions

**Naho Kurahashi**: Writing – original draft, conceptualization, methodology, data curation, investigation, visualization, formal analysis, project administration. **Maximilian Schiffer**: methodology, writing – original draft, writing – review and editing, investigation, visualization, formal analysis, software, resources. **Cedric Kreusel**: investigation, writing – review and editing. **Christian Tückmantel**: methodology, software, resources, writing – review and editing, investigation. **Ahmed Kadid**: investigation, formal analysis, visualization, writing – review and editing. **Habib Ur Rehman**: investigation, visualization, writing – review and editing. **Florian Gaschler**: software, methodology, data curation, investigation, formal analysis, visualization. **René Anfang**: investigation, methodology, software, formal analysis. **Christopher Knoth**: methodology, software, formal analysis, investigation, data curation, visualization. **Maximilian Buchmüller**: investigation, writing – original draft, writing – review and editing, methodology, data curation, formal analysis, visualization, supervision, conceptualization, project administration. **Patrick Görrn**: funding acquisition, writing – original draft, writing – review and editing, project administration, supervision, methodology, investigation, conceptualization. **Thomas Riedl**: funding acquisition, conceptualization, writing – original draft, writing – review and editing, project administration, supervision.

## Funding

This work was supported by the German Research Foundation (DFG) Hiper‐Lase (RI 1551/18‐1), DFG 2D‐FASOL (Rl 1551/22‐1), DFG ALSATIAN (RI 1551/23‐1); the German Federal Ministry of Research, Technology and Space NEPOMUQ (13N17112), and Alexander von Humboldt Stiftung (Humboldt Research Fellowship Programme for Postdocs).

## Conflicts of Interest

The authors declare no conflicts of interest.

## Supporting information




**Supporting File**: advs78163‐sup‐0001‐SuppMat.docx.

## Data Availability

Data is available on request from the authors.

## References

[1] I. L. Braly , D. W. Dequilettes , L. M. Pazos‐Outón , et al., “Hybrid Perovskite Films Approaching the Radiative Limit With Over 90% Photoluminescence Quantum Efficiency,” Nature Photonics 12, no. 6 (2018): 355–361, 10.1038/s41566-018-0154-z.

[2] W. B. Gunnarsson , K. Roh , L. Zhao , et al., “Toward Nonepitaxial Laser Diodes,” Chemical Reviews 123, no. 12 (2023): 7548–7584, 10.1021/acs.chemrev.2c00721. 37219995

[3] A. Cherepakhin , A. Zhizhchenko , D. Khmelevskaia , L. Logunov , A. Kuchmizhak , and S. Makarov , “Advanced Laser Nanofabrication Technologies for Perovskite Photonics,” Advanced Optical Materials 12, no. 13 (2024): 2302782, 10.1002/adom.202302782.

[4] Y. Gao , Q. Cai , Y. He , et al., “Highly Efficient Blue Light‐emitting Diodes Based on Mixed‐halide Perovskites With Reduced Chlorine Defects,” Science Advances 10, no. 29 (2024): 5645, 10.1126/sciadv.ado5645. PMC466955 39018409

[5] S. Q. Sun , J. W. Tai , W. He , et al., “Enhancing Light Outcoupling Efficiency via Anisotropic Low Refractive Index Electron Transporting Materials for Efficient Perovskite Light‐Emitting Diodes,” Advanced Materials 36, no. 24 (2024): 2400421, 10.1002/adma.202400421. 38430204

[6] S.‐C. Feng , Y. Shen , X.‐M. Hu , et al., “Efficient and Stable Red Perovskite Light‐Emitting Diodes via Thermodynamic Crystallization Control,” Advanced Materials 36, no. 44 (2024): 2410255, 10.1002/adma.202410255. 39223930

[7] M. Li , Y. Yang , Z. Kuang , et al., “Acceleration of Radiative Recombination for Efficient Perovskite LEDs,” Nature 630, no. 8017 (2024): 631–635, 10.1038/s41586-024-07460-7. PMC11186751 38811739

[8] B. R. Sutherland and E. H. Sargent , “Perovskite Photonic Sources,” Nature Photonics 10, no. 5 (2016): 295–302, 10.1038/nphoton.2016.62.

[9] S. Chen and A. Nurmikko , “Stable Green Perovskite Vertical‐Cavity Surface‐Emitting Lasers on Rigid and Flexible Substrates,” ACS Photonics 4 (2017): 2486–2494.

[10] S. Chen , C. Zhang , J. Lee , J. Han , and A. Nurmikko , “High‐ Q , Low‐Threshold Monolithic Perovskite Thin‐Film Vertical‐Cavity Lasers,” Advanced Materials 29, no. 16 (2017): 1604781, 10.1002/adma.201604781. 28211117

[11] Y. Shi , X. Deng , Y. Gan , L. Xu , Q. Zhang , and Q. Xiong , “Ten Years of Perovskite Lasers,” Advanced Materials 37, no. 25 (2025): 2413559, 10.1002/adma.202413559. 39828626

[12] L. Zhao , K. Roh , S. Kacmoli , et al., “Nanosecond‐Pulsed Perovskite Light‐Emitting Diodes at High Current Density,” Advanced Materials 33, no. 44 (2021): 2104867, 10.1002/adma.202104867. 34477263

[13] K. Elkhouly , I. Goldberg , N. Annavarapu , et al., “Intense Electrical Pulsing of Perovskite Light Emitting Diodes Under Cryogenic Conditions,” Advanced Optical Materials 10, no. 15 (2022): 2200024, 10.1002/adom.202200024.

[14] H. Kim , K. Roh , J. P. Murphy , et al., “Optically Pumped Lasing From Hybrid Perovskite Light‐Emitting Diodes,” Advanced Optical Materials 8, no. 1 (2020): 1901297, 10.1002/adom.201901297.

[15] C. Cho , T. Antrack , M. Kroll , et al., “Electrical Pumping of Perovskite Diodes: Toward Stimulated Emission,” Advanced Science 8, no. 17 (2021): 2101663, 10.1002/advs.202101663. PMC8425921 34240575

[16] K. Elkhouly , I. Goldberg , X. Zhang , et al., “Electrically Assisted Amplified Spontaneous Emission in Perovskite Light‐emitting Diodes,” Nature Photonics 18, no. 2 (2024): 132–138, 10.1038/s41566-023-01341-7.

[17] A. J. Grede , R. Cawthorn , L. Zhao , et al., “Electrically Assisted Lasing in Metal Halide Perovskite Semiconductors,” ACS Photonics 11, no. 5 (2024): 1851–1856, 10.1021/acsphotonics.4c00642.

[18] C. Zou , Z. Ren , K. Hui , et al., “Electrically Driven Lasing From a Dual‐cavity Perovskite Device,” Nature 645, no. 8080 (2025): 369–374, 10.1038/s41586-025-09457-2. PMC12422965 40866697

[19] A. P. Pushkarev , D. Khmelevskaia , I. A. Matchenya , et al., “Non‐epitaxial Perovskite Polariton Laser Diode Operating Under Direct Current,” Nature 656, no. 8126 (2026): 80–85, 10.1038/s41586-026-10824-w. 42486972

[20] Y. Jiang , M. Cui , S. Li , et al., “Reducing the Impact of Auger Recombination in Quasi‐2D Perovskite Light‐Emitting Diodes,” Nature Communications 12 (2021): 336. 10.1038/s41467-020-20555-9 PMC7804015 33436618

[21] C. Bi , Z. Yao , J. Hu , et al., “Suppressing Auger Recombination of Perovskite Quantum Dots for Efficient Pure‐Blue‐Light‐Emitting Diodes,” ACS Energy Letters 8, no. 1 (2023): 731–739, 10.1021/acsenergylett.2c02613.

[22] M. R. Leyden , S. Terakawa , T. Matsushima , et al., “Distributed Feedback Lasers and Light‐Emitting Diodes Using 1‐Naphthylmethylamnonium Low‐Dimensional Perovskite,” ACS Photonics 6 (2019): 460–466.

[23] H. Li , X. Zhu , D. Zhang , et al., “Thermal Management Towards Ultra‐bright and Stable Perovskite Nanocrystal‐based Pure Red Light‐emitting Diodes,” Nature Communications 15 (2024): 1. 10.1038/s41467-024-50634-0 PMC11297279 39095426

[24] T. J. S. Evans , A. Schlaus , Y. Fu , et al., “Continuous‐Wave Lasing in Cesium Lead Bromide Perovskite Nanowires,” Advanced Optical Materials 6, no. 2 (2018): 1700982, 10.1002/adom.201700982.

[25] C. Qin , A. S. D. Sandanayaka , C. Zhao , et al., “Stable Room‐Temperature Continuous‐wave Lasing in Quasi‐2D Perovskite Films,” Nature 585, no. 7823 (2020): 53–57, 10.1038/s41586-020-2621-1. 32879501

[26] J. Wang , N. Wang , Y. Jin , et al., “Interfacial Control toward Efficient and Low‐Voltage Perovskite Light‐Emitting Diodes,” Advanced Materials 27, no. 14 (2015): 2311–2316, 10.1002/adma.201405217. 25708283

[27] Y. Chen , X. Zuo , Y. He , et al., “Dual Passivation of Perovskite and SnO_2_ for High‐Efficiency MAPbI_3_ Perovskite Solar Cells,” Advanced Science 8, no. 5 (2021): 2001466, 10.1002/advs.202001466. PMC7927604 33717834

[28] M. Du , S. Zhao , L. Duan , et al., “Surface Redox Engineering of Vacuum‐Deposited NiOx for Top‐Performance Perovskite Solar Cells and Modules,” Joule 6 (2022): 1931.

[29] H. Baqandwan , S. A. Mohamad Samsuri , M. M. Halim , and M. Z. Pakhuruddin , “Nanostructured TiO_2_ Thin Films Sputtered at Room Temperature as Electron Transport Layer for Flexible Perovskite Solar Cell Applications: Impact of Varying RF Power,” Physica B: Condensed Matter 679 (2024): 415788, 10.1016/j.physb.2024.415788.

[30] N. K. Kim , Y. H. Min , S. Noh , et al., “Investigation of Thermally Induced Degradation in CH_3_NH_3_PbI_3_ Perovskite Solar Cells Using in‐situ Synchrotron Radiation Analysis,” Scientific Reports 7, no. 1 (2017): 4645, 10.1038/s41598-017-04690-w. PMC5498566 28680138

[31] X. Qiu , B. Cao , S. Yuan , et al., “From Unstable CsSnI_3_ to Air‐Stable Cs_2_SnI_6_: A Lead‐Free Perovskite Solar Cell Light Absorber with Bandgap of 1.48 eV and High Absorption Coefficient,” Solar Energy Materials and Solar Cells 159 (2017): 227–234, 10.1016/j.solmat.2016.09.022.

[32] N. Kurahashi , M. Runkel , C. Kreusel , et al., “Distributed Feedback Lasing in Thermally Imprinted Phase‐Stabilized CsPbI_3_ Thin Films,” Advanced Functional Materials 34, no. 45 (2024): 2405976, 10.1002/adfm.202405976.

[33] J. Li , X. Shan , S. G. R. Bade , et al., “Single‐Layer Halide Perovskite Light‐Emitting Diodes With Sub‐Band Gap Turn‐On Voltage and High Brightness,” The Journal of Physical Chemistry Letters 7, no. 20 (2016): 4059–4066, 10.1021/acs.jpclett.6b01942. 27689966

[34] S. B. Cho , C. X. Li , D. H. Kwak , Y. N. Choi , and I. K. Park , “Enhanced Emission Efficiency and Stability of Blue‐Emitting Quasi‐2D Perovskites via Strain‐Modulating Lewis Base Addition,” ACS Applied Materials & Interfaces 17, no. 41 (2025): 57273–57283, 10.1021/acsami.5c12740. 41032863

[35] M. Marchal , E. Dimopoulos , K. Spiegelhauer , et al., “Carrier Transport in Electrically‐Driven Photonic Crystal Membrane Lasers,” Laser & Photonics Reviews 20 (2025): 01579.

[36] K. Iga , “Surface‐Emitting Laser‐Its Birth and Generation of New Optoelectronics Field,” IEEE Journal of Selected Topics in Quantum Electronics 6, no. 6 (2000): 1201–1215, 10.1109/2944.902168.

[37] J. Baker , S. Gillgrass , C. P. Allford , et al., “VCSEL Quick Fabrication for Assessment of Large Diameter Epitaxial Wafers,” IEEE Photonics Journal 14, no. 3 (2022): 1–10, 10.1109/JPHOT.2022.3169032.

[38] T. S. Sherkar , C. Momblona , L. Gil‐Escrig , et al., “Recombination in Perovskite Solar Cells: Significance of Grain Boundaries, Interface Traps, and Defect Ions,” ACS Energy Letters 2, no. 5 (2017): 1214–1222, 10.1021/acsenergylett.7b00236. PMC5438194 28540366

[39] Z. Chu , M. Yang , P. Schulz , et al., “Impact of Grain Boundaries on Efficiency and Stability of Organic‐Inorganic Trihalide Perovskites,” Nature Communications 8 (2017): 2230. 10.1038/s41467-017-02331-4 PMC5738431 29263379

[40] X. Li , W. Liu , Y. Song , et al., “Two‐photon‐pumped High‐Quality, Single‐Mode Vertical Cavity Lasing Based on Perovskite Monocrystalline Films,” Nano Energy 68 (2020): 104334, 10.1016/j.nanoen.2019.104334.

[41] H. Zhang , C. Zou , Y. Chen , et al., “Continuous‐Wave Vertical Cavity Surface‐Emitting Lasers Based on Single Crystalline Lead Halide Perovskites,” Advanced Optical Materials 9, no. 13 (2021): 2001982, 10.1002/adom.202001982.

[42] S. Aoyagi , Z. Su , G. Weng , et al., “Single‐crystal CsPbBr _3_ ‐Based Vertical Cavity Surface Emitting Laser,” Optics Letters 50, no. 2 (2025): 702, 10.1364/OL.547212. 39815597

[43] N. Pourdavoud , S. Wang , A. Mayer , et al., “Photonic Nanostructures Patterned by Thermal Nanoimprint Directly Into Organo‐Metal Halide Perovskites,” Advanced Materials 29, no. 12 (2017): 1605003, 10.1002/adma.201605003. 28102599

[44] N. Pourdavoud , A. Mayer , M. Buchmüller , et al., “Distributed Feedback Lasers Based on MAPbBr_3_ ,” Advanced Materials Technologies 3, no. 4 (2018): 1700253, 10.1002/admt.201700253.

[45] N. Pourdavoud , T. Haeger , A. Mayer , et al., “Room‐Temperature Stimulated Emission and Lasing in Recrystallized Cesium Lead Bromide Perovskite Thin Films,” Advanced Materials 31, no. 39 (2019): 1903717, 10.1002/adma.201903717. 31402527

[46] K. L. Shaklee and R. F. Leheny , “Direct Determination of Optical Gain in Semiconductor Crystals,” Applied Physics Letters 18, no. 11 (1971): 475–477, 10.1063/1.1653501.

[47] A. L. Alvarado‐Leaños , D. Cortecchia , G. Folpini , A. R. Srimath Kandada , and A. Petrozza , “Optical Gain of Lead Halide Perovskites Measured via the Variable Stripe Length Method: What We Can Learn and How to Avoid Pitfalls,” Advanced Optical Materials 9, no. 18 (2021): 2001773, 10.1002/adom.202001773.

[48] K. Roh , L. Zhao , and B. P. Rand , “Tuning Laser Threshold Within the Large Optical Gain Bandwidth of Halide Perovskite Thin Films,” ACS Photonics 8, no. 8 (2021): 2548–2554, 10.1021/acsphotonics.1c00910.

[49] N. Annavarapu , I. Goldberg , A. Papadopoulou , et al., “Scattered Emission Profile Technique for Accurate and Fast Assessment of Optical Gain in Thin Film Lasing Materials,” ACS Photonics 10 (2023): 1583.

[50] M. Aellen and D. J. Norris , “Understanding Optical Gain: Which Confinement Factor Is Correct?,” ACS Photonics 9, no. 11 (2022): 3498–3505, 10.1021/acsphotonics.2c01222.

[51] J. Ye , A. Ren , L. Dai , et al., “Direct Linearly Polarized Electroluminescence From Perovskite Nanoplatelet Superlattices,” Nature Photonics 18, no. 6 (2024): 586–594, 10.1038/s41566-024-01398-y.

[52] Z. Yang , A. Surrente , K. Galkowski , et al., “Impact of the Halide Cage on the Electronic Properties of Fully Inorganic Cesium Lead Halide Perovskites,” ACS Energy Letters 2, no. 7 (2017): 1621–1627, 10.1021/acsenergylett.7b00416.

[53] D. L. Gau , I. Galain , I. Aguiar , and R. E. Marotti , “Origin of Photoluminescence and Experimental Determination of Exciton Binding Energy, Exciton‐phonon Interaction, and Urbach Energy in γ‐CsPbI_3_ Nanoparticles,” Journal of Luminescence 257 (2023): 119765, 10.1016/j.jlumin.2023.119765.

[54] W. W. Chow and S. W. Koch , Semiconductor‐Laser Fundamentals (Springer Nature, 1999), 10.1007/978-3-662-03880-2.

[55] M. Meudt , C. Bogiadzi , K. Wrobel , and P. Görrn , “Hybrid Photonic–Plasmonic Bound States in Continuum for Enhanced Light Manipulation,” Advanced Optical Materials 8, no. 20 (2020): 2000898, 10.1002/adom.202000898.

[56] K. H. Wang , L. C. Li , M. Shellaiah , and K. W. Sun , “Structural and Photophysical Properties of Methylammonium Lead Tribromide (MAPbBr3) Single Crystals,” Scientific Reports 7, no. 1 (2017): 13643, 10.1038/s41598-017-13571-1. PMC5651898 29057892

[57] J. Shi , H. Zhang , Y. Li , et al., “Identification of High‐temperature Exciton States and Their Phase‐dependent Trapping Behaviour in Lead Halide Perovskites,” Energy & Environmental Science 11, no. 6 (2018): 1460–1469, 10.1039/C7EE03543H.

[58] M. Li , P. Huang , and H. Zhong , “Current Understanding Of Band‐edge Properties of Halide Perovskites: Urbach Tail, Rashba Splitting, and Exciton Binding Energy,” Journal of Physical Chemistry Letters 14 (2023): 5. 10.1021/acs.jpclett.2c03525 36749031

[59] M. Saba , M. Cadelano , D. Marongiu , et al., “Correlated Electron–Hole Plasma in Organometal Perovskites,” Nature Communications 5, no. 1 (2014): 5049, 10.1038/ncomms6049. 25266869

[60] W. W. Chow , S. W. Koch , and M. Sargent , Semiconductor‐Laser Physics (Springer Nature, 1994), 10.1007/978-3-642-61225-1.

[61] C. Y. Huang , C. Zou , C. Mao , et al., “CsPbBr_3_ Perovskite Quantum Dot Vertical Cavity Lasers with Low Threshold and High Stability,” ACS Photonics 4, no. 9 (2017): 2281–2289, 10.1021/acsphotonics.7b00520.

[62] Y. Li , J. Roger , I. Allegro , et al., “Lasing from Laminated Quasi‐2D/3D Perovskite Planar Heterostructures,” Advanced Functional Materials 32, no. 27 (2022): 2200772, 10.1002/adfm.202200772.

[63] R. An , F. Zhang , X. Zou , et al., “Photostability and Photodegradation Processes in Colloidal CsPbI_3_ Perovskite Quantum Dots,” ACS Applied Materials & Interfaces 10, no. 45 (2018): 39222–39227, 10.1021/acsami.8b14480. 30350934

[64] M. S. Alias , Z. Liu , A. Al‐atawi , T. K. Ng , T. Wu , and B. S. Ooi , “Continuous‐wave Optically Pumped Green Perovskite Vertical‐cavity Surface‐emitter,” Optics Letters 42, no. 18 (2017): 3618, 10.1364/OL.42.003618. 28914916

[65] R. P. Sarzala , L. Piskorski , T. Czyszanowski , and M. Dems , “Influence of Various Bottom DBR Designs on the Thermal Properties of Blue Semiconductor‐Metal Subwavelength‐Grating VCSELs,” Materials 12, no. 19 (2019): 3235, 10.3390/ma12193235. PMC6803851 31581660

[66] H. Yu , X. Cheng , Y. Wang , et al., “Waterproof Perovskite‐Hexagonal Boron Nitride Hybrid Nanolasers With Low Lasing Thresholds and High Operating Temperature,” ACS Photonics 5, no. 11 (2018): 4520–4528, 10.1021/acsphotonics.8b00977.

[67] C. Steinberg , K. Dhima , D. Blenskens , et al., “A Scalable Anti‐sticking Layer Process via Controlled Evaporation,” Microelectronic Engineering 123 (2014): 4–8, 10.1016/j.mee.2014.04.002.

[68] A. Mayer , S. Moellenbeck , K. Dhima , S. Wang , and H.‐C. Scheer , “Mechanical Characterization of a Piezo‐Operated Thermal Imprint System,” Journal of Vacuum Science & Technology B, Nanotechnology and Microelectronics: Materials, Processing, Measurement, and Phenomena 29 (2011): 06FC13.

[69] M. Papenheim , C. Steinberg , K. Dhima , S. Wang , and H.‐C. Scheer , “Flexible Composite Stamp for Thermal Nanoimprint Lithography Based on OrmoStamp,” Journal of Vacuum Science & Technology B, Nanotechnology and Microelectronics: Materials, Processing, Measurement, and Phenomena 33, no. 6 (2015): 06F601, 10.1116/1.4929885.

