# Supplementary material for: Low Threshold Vertical Cavity Surface Emitting Lasers Based on CsPbI3 Thin Films
Source: Adv Sci (Weinh). 2026 Oct 12:e78163. Online ahead of print. doi: 10.1002/advs.78163 (PMC13658881; doi:10.1002/advs.78163)
Supplement: Supplementary file 1 [file ADVS-9999-e78163-s001.docx]

Supporting Information

**Low Threshold Vertical Cavity Surface Emitting Lasers Based on CsPbI_3_ Thin Films**

*Naho Kurahashi,* Maximilian Schiffer, Cedric Kreusel, Christian Tückmantel, Ahmed Kadid, Habib Ur Rehman, Florian Gaschler, René Anfang, Christopher Knoth, Maximilian Buchmüller, Patrick Görrn, and Thomas Riedl**

N. Kurahashi, M. Schiffer, C. Kreusel, C. Tückmantel, A. Kadid, H. Ur Rehman, T. Riedl

Institute of Electronic Devices, University of Wuppertal, Rainer-Gruenter-Str. 21, 42119 Wuppertal, Germany

and

Wuppertal Center for Smart Materials & Systems (CM@S), University of Wuppertal, 42119 Wuppertal, Germany

E-mail: [t.riedl@uni-wuppertal.de](mailto:t.riedl@uni-wuppertal.de), [kurahashi@uni-wuppertal.de](mailto:kurahashi@uni-wuppertal.de)

F. Gaschler, R. Anfang, C. Knoth, M. Buchmüller, P. Görrn

Chair of Large Area Optoelectronics, University of Wuppertal, Rainer-Gruenter-Str. 21, 42119 Wuppertal, Germany

and

Wuppertal Center for Smart Materials & Systems (CM@S), University of Wuppertal, 42119 Wuppertal, Germany

**Distributed Bragg reflector (DBR)**

**Figure S1.** Transmission spectrum of the DBR used to prepare the vertical-cavity surface-emitting lasers (VCSELs) in this work.^[1]^

**Characterization of CsPbI_3_: poly(vinylpyrrolidone) (PVP) films on the DBR**

**Figure S2.** Top-view scanning electron microscope (SEM) images of (a) pristine and (b) pressed CsPbI_3_:PVP films. (c) X-ray diffractograms of pristine (black line) and pressed (red line) CsPbI_3_:PVP films. The assignment of the peak positions was taken from Ref.[2]. Both patterns are consistent with the *γ*-phase CsPbI_3_ reported in our previous work.^[3]^

**Photoluminescence (PL) of the pressed CsPbI_3_:PVP films on the DBR**

**Figure S3.** PL spectra of the pressed 100 nm- and 60 nm-thick CsPbI_3_:PVP films on the DBR. The side peak exhibits a wavelength shift with varying film thickness, suggesting that the feature is related with an optical resonance (potentially related to the sequence DBR/perovskite/air) rather than a secondary material phase.

**Optical confinement in perovskite thin films**

The optical confinement factor (*Г*) of the waveguide was determined through numerical analysis using the transfer matrix method. Specifically, the simulations were performed for the fundamental transverse electric (TE_0_) mode, which corresponds directly to the TE-polarized edge emission selected during the variable stripe length measurements. The waveguide structure was modeled as an asymmetric slab consisting of an air superstrate (n = 1.00), a perovskite active layer (n = 2.4), and a 1500 nm-thick SiO_2_ cladding layer (n = 1.46). The confinement factor was determined by analyzing the calculated electric field distributions for each film thickness at the emission wavelength of 720 nm.

**Figure S4.** Calculated the electric field distribution for the TE_0_ mode and refractive index profile of (a) the 100 nm-thick and (b) the 60 nm-thick samples.

**Exciton binding energy of the pressed CsPbI_3_:PVP films**

The exciton binding energy (*E_b_*) of the CsPbI_3_:PVP films was estimated from the room-temperature absorption spectra using Elliott model,^[4]^ which accounts for both discrete excitonic transitions and continuum band-to-band absorption. Due to thermal and spectral broadening at room temperature, the extracted *E_b_* is subject to uncertainty and is therefore reported here as an approximate value. For the 100 nm-thick film, the excitonic resonance (*E_0_*) is centered at 1.758 eV, while the continuum onset corresponding to the bandgap (*E_g_*) is located at 1.777 eV, resulting in an estimated *E_b_* of 18 meV (with a numerical fitting uncertainty of ±0.4 meV).

To examine the thickness dependence, the same analysis was performed for a 60 nm-thick film. The extracted parameters (*E_0_* = 1.762 eV, *E_g_* = 1.778 eV, and *E_b_* = 16±0.3 meV) remain essentially consistent with the results of 100 nm-thick film. Notably, the 60 nm-thick film exhibits a doubling in excitonic resonance amplitude as that of 100 nm-thick film as well as a smaller bandwidth (40 and 47 meV for 60 and 100 nm-thick films, respectively).

**Figure S5.** Absorption spectrum with Elliott’s fits (black dotted curves) consisting of excitonic peaks (blue dotted curves) and continuum contribution (green dotted curves) of (a) 100 nm-thick and (b) 60 nm-thick pressed CsPbI_3_:PVP film.

**Temperature-dependent PL**

**Figure S6.** Temperature-dependent PL intensities of 100 nm-thick and 60 nm-thick pressed CsPbI_3_:PVP film. The solid line is fit based on the Arrhenius equation.^[5]^

**Thickness of CsPbI_3_:PVP film on the DBR after planar hot pressing (PHP)**

**Figure S7.** Cross-sectional SEM image of CsPbI_3_:PVP film on the DBR after PHP. The sample was prepared by delaminating the top DBR from the fabricated VCSEL structure after the PHP process and then mechanically cleaving it to expose the cross section. The image confirms a highly uniform active layer thickness of 60±1.6 nm (determined from 15 points across the cross-section) in the optimized device. Elemental analysis by energy-dispersive X-ray spectroscopy (EDX) confirmed the presence of SiO_2_ and Ta_2_O_5_ in the DBR stack, with the brighter and darker layers corresponding to Ta_2_O_5_ and SiO_2_, respectively, based on SEM image contrast.

**Rigorous coupled wave analysis (RCWA) simulations**

For the RCWA simulations, we first recorded a cross-sectional SEM image of one of the DBR and extracted a horizontally averaged grayscale intensity profile, revealing a well-defined ABAB-type nanolaminate structure (see Figure S8). Elemental analysis by EDX identified the alternating layers as SiO_2_ and Ta_2_O_5_, with the brighter layers corresponding to Ta_2_O_5_ and the darker to SiO_2_. The SiO_2_ substrate is visible at the bottom of the image in S5, while the upper region shows the mirror surface, where charging effects are observed around the edge of the cleaved structure.

**Figure S8.** Cross-sectional SEM image of the DBR with corresponding horizontally averaged grayscale profile.

Using the grayscale profile shown in red in Figure S8, each of the 50 layers of the nanolaminate was assigned an individual layer thickness, with the interfaces between adjacent layers defined at the average between local intensity minima and maxima. These film thicknesses together with complex refractive indices for the bulk materials from Palik^[6]^ were then used for RCWA simulations. Based on this reconstruction, the simulated transmission spectra already showed good agreement with the experimental data. However, further optimization of the structure was required to accurately reproduce the resonance positions of the complete VCSEL stack.

To reliably identify resonance positions, we evaluated the derivative of the simulated phase of transmitted light, as the phase exhibits pronounced variations in the proximity of a resonance. We then implemented a constrained optimization procedure in which the thicknesses of all SiO_2_ layers and all Ta_2_O_5_ layers were independently varied by two variation factors. This strategy accounts for uncertainties in thickness extraction from SEM data as well as for assumptions made in the simulation, such as perfectly smooth, homogeneous layers and abrupt layer interfaces, while keeping the refractive indices fixed at their bulk values. Allowing thickness adjustments of less than 10% yielded good agreement between simulated and measured resonance positions for perovskite thicknesses of 60 nm and 80 nm (see Figure S9). The final thickness list of each of the layers of the DBR can be found in the following Table S1.

**Figure S9.** Derivative of the phase of transmitted light indicating the positions of resonances in the VCSEL stack.

**Table S1.** Layer thickness list of the optimized DBR.

| Layer No. | Thickness (nm) | Layer No. | Thickness (nm) | Layer No. | Thickness (nm) |
| --- | --- | --- | --- | --- | --- |
| 1 | 59.1 | 18 | 83.9 | 35 | 117.6 |
| 2 | 49.2 | 19 | 164.6 | 36 | 79.8 |
| 3 | 166.5 | 20 | 98.1 | 37 | 120.5 |
| 4 | 72.2 | 21 | 179.2 | 38 | 74.7 |
| 5 | 185.7 | 22 | 82.7 | 39 | 124.4 |
| 6 | 83.1 | 23 | 152.2 | 40 | 77.6 |
| 7 | 178.3 | 24 | 77.8 | 41 | 144.2 |
| 8 | 104.0 | 25 | 137.0 | 42 | 182.8 |
| 9 | 148.6 | 26 | 67.6 | 43 | 149.3 |
| 10 | 84.8 | 27 | 125.5 | 44 | 65.7 |
| 11 | 193.6 | 28 | 72.1 | 45 | 132.7 |
| 12 | 78.3 | 29 | 138.5 | 46 | 70.9 |
| 13 | 182.0 | 30 | 78.0 | 47 | 126.9 |
| 14 | 80.8 | 31 | 127.7 | 48 | 80.5 |
| 15 | 134.7 | 32 | 75.2 | 49 | 128.1 |
| 16 | 68.0 | 33 | 138.9 | 50 | 102.8 |
| 17 | 138.4 | 34 | 82.0 |  |  |

For both perovskite thicknesses, two resonances are found within the relevant spectral range with positions close to the experimental values (deviations in the range of a few nanometers). Upon increasing the perovskite thickness from 60 nm to 80 nm, both resonances shift spectrally, consistent with the experimentally observed shift in the laser emission wavelength.

In close proximity of these resonance positions, the eigenmodes could be numerically calculated. Representative electric field profiles for the 60 nm perovskite layer are shown in Figure S10. The shorter-wavelength resonance at 716.3 nm exhibits a pronounced electric field intensity maximum within the perovskite layer in the center. In contrast, the longer-wavelength resonance at 741.4 nm has an electric field intensity minimum within the perovskite region, suggesting that the resonance at 716.3 nm could be favored for lasing, also considering their overlap with the gain spectrum of the material.

**Figure S10.** Simulated electric field intensity profiles for the two modes shown in Figure S9 for a perovskite thickness of 60 nm.

Although minor deviations of a few nanometers in the resonance positions remain, the overall agreement between simulation and experiment demonstrates that the reconstructed stack consisting of 101 layers and the RCWA simulations capture the optical behavior of the sample, particularly the resonance shift induced by variations in perovskite thickness, which could not be explained by a Fabry-Pérot cavity model.

**Lasing characteristics of VCSEL with 80 nm-thick CsPbI_3_:PVP film**

**Figure S11.** (a) Emission spectrum (at a fluence of 3.8 mJ cm^−2^) obtained from a VCSEL with an 80 nm-thick CsPbI_3_:PVP film and (b) output characteristics upon optical pumping with the determined lasing threshold. The VCSEL exhibited only occasional lasing with an extremely high threshold (3.0 mJ cm^−2^) because the cavity resonance lies near the edge of the positive gain region in the gain spectrum.

**Q factor of VCSEL with 100 nm-thick CsPbI_3_:PVP layer**

**Figure S12.** Cavity mode spectrum obtained from a VCSEL with a 100 nm-thick CsPbI_3_:PVP layer. The resonance peak centered at 753.1 nm exhibits a FWHM of 1.77 nm, corresponding to a Q factor of ~425. Note that this device did not exhibit lasing because the cavity resonance falls outside the positive optical gain region.

**Power-dependent PL**

**Figure S13.** PL intensity (*I_PL_*) measured at varied excitation power (*P*) for (a)100 nm-thick and (b) 60 nm-thick pressed CsPbl_3_:PVP films. The exponent *k* has been determined by fitting *I_PL_*~*P^k^* to the data (red line).

**Gain charactraistics of 60 nm-thick CsPbI_3_:PVP film**

**Figure S14.** (a) Amplified spontaneous emission (ASE) characteristics of the pressed 60 nm-thick CsPbI_3_:PVP film and (b) corresponding output characteristics upon optical pumping with determined threshold. (c) Gain spectra of pressed CsPbI_3_: PVP film with a thickness of 60 nm on Si/SiO_2_ extracted by variable stripe length method and (d) corresponding gain slope.

**Table S2.** Summary of key parameters in representative thin film based perovskite lasers operated at room temperature.

| Material | Cavity | Pump  (nm) | Pulse  (ns) | Emission  (nm) | Threshold  (μJ cm^-2^) | FWHM (nm) | Ref. |
| --- | --- | --- | --- | --- | --- | --- | --- |
| CH_3_NH_3_PbI_3_ | VCSEL | 532 | 0.34 | 778.4 | 7.6 ± 0.6 | 0.24 | [7] |
| FAPbBr_3_ | VCSEL | 355 | 0.34 | 552.4 | 18.3 ± 1.5 | 0.39 | [8] |
| CH_3_NH_3_PbBr_3_ | DFB | 532 | 0.3 | 555.3 | 3.4 | 0.14 | [9] |
| CsPbBr_3_ | DFB | 355 | 0.3 | 538 | 10 | 0.12 | [10] |
| CsPbBr_3_ | VCSEL | 355 | 0.3 | 538 | 2.2 | 0.07 | [10] |
| CsPbI_3_ | DFB | 532 | 0.3 | 719 | 45 | 0.19 | [3] |
| CsPbI_3_ | VCSEL | 355 | 0.3 | 720.4 | 2.8 | 0.16 | **This work** |

**References**

[1]	https://www.thorlabs.com/dichroic-filters?tabName=Graphs.

[2]	C. Dong *et al.*, Adv. Funct. Mater. **33**, (2023).

[3]	N. Kurahashi *et al.*, Adv. Funct. Mater. (2024).

[4]	Q. Chang *et al.*, Communications Physics **5**, 1 (2022).

[5]	N. Droseros *et al.*, ACS Energy Lett. **3**, 1458 (2018).

[6]	E. D. Palik, Handbook of Optical Constants of Solids **1**, 1 (2012).

[7]	S. Chen *et al.*, Advanced Materials **29**, 1604781 (2017).

[8]	S. Chen and A. Nurmikko, ACS Photonics **4**, 2486 (2017).

[9]	N. Pourdavoud *et al.*, Adv. Mater. Technol. **3**, 1700253 (2018).

[10]	N. Pourdavoud *et al.*, Advanced Materials **31**, 1903717 (2019).
